# Mental Health and Other Factors Associated with COVID-19 Vaccination Intention toward Children of Military Parents in Lambayeque, Peru

**DOI:** 10.1155/2024/8873387

**Published:** 2024-06-21

**Authors:** Mario J. Valladares-Garrido, Aldo Alvarez-Risco, Annel B. Rojas-Alvarado, Cinthia Karina Picón-Reátegui, Franccesca Dawson Aguila, Shyla Del-Aguila-Arcentales, Neal M. Davies, Virgilio E. Failoc-Rojas, César Johan Pereira-Victorio, Danai Valladares Garrido, Víctor J. Vera-Ponce, Jaime A. Yáñez

**Affiliations:** ^1^Escuela de Medicina, Universidad Cesar Vallejo, Piura, Peru; ^2^Oficina de Epidemiología, Hospital Regional Lambayeque, Chiclayo, Peru; ^3^Universidad Tecnológica del Perú, Lima, Peru; ^4^Escuela de Medicina, Universidad Privada Antenor Orrego, Piura, Peru; ^5^Universidad de San Martín de Porres, Facultad de Medicina Humana, Chiclayo, Peru; ^6^Escuela de Posgrado, Universidad San Ignacio de Loyola, Lima, Peru; ^7^Faculty of Pharmacy & Pharmaceutical Sciences, University of Alberta, Edmonton, AB T6G 2H1, Canada; ^8^Research Unit for Generation and Synthesis Evidence in Health, Universidad San Ignacio de Loyola, Lima, Peru; ^9^School of Medicine, Universidad Continental, Lima, Peru; ^10^Oficina de Salud Ocupacional, Hospital Santa Rosa, Piura, Peru; ^11^Instituto de Investigación de Enfermedades Tropicales, Universidad Nacional Toribio Rodríguez de Mendoza de Amazonas, Chachapoyas, Amazonas, Peru; ^12^Facultad de Medicina (FAMED), Universidad Nacional Toribio Rodríguez de Mendoza de Amazonas (UNTRM), Amazonas, Peru; ^13^Universidad Peruana de Ciencias Aplicadas, Facultad de Educación, Carrera de Educación y Gestión del Aprendizaje, Lima, Peru

## Abstract

There is evidence that vaccine acceptability is strongly associated with mental health. However, no studies assessing intention to vaccinate (ITV) intention toward children of military parents have been documented. The current research aimed to establish the prevalence and factors of ITV children against COVID-19 in military parents in Lambayeque-Peru, 2021. Analysis was conducted with the dependent variable ITV children reported by military parents. The independent variables were history of mental health, searching for mental health support, food insecurity, resilience, anxiety, depression, burnout, posttraumatic stress, and suicidal risk. Prevalence ratios and 95% confidence intervals were estimated. Of 201 military personnel evaluated, 92.5% were male, 82.5% were of the Catholic faith, and the median age was 40.9% of respondents reported seeking mental health help during the COVID-19 pandemic. It was reported anxiety (20.3%), depression (6.5%), and posttraumatic stress disorder (6.5%). Most reported ITV in children against COVID-19 (93%). In the multiple models, we found that Catholics had a 23% higher prevalence of ITV in the children where PR = prevalence ratios and CI = confidence intervals (PR = 1.23; 95% CI: 1.01–1.50). Likewise, seeking mental health support increased the prevalence of ITV by 8% (PR = 1.08; 95% CI: 1.00–1.15). Seeking mental health support and belonging to the Catholic faith had a higher ITV of children of Peruvian military personnel. Finding mental health support, experiencing burnout syndrome, having a relative who suffers from mental health problems, and being part of the Catholic religion were associated with a higher willingness to immunize the children of Peruvian military members.

## 1. Introduction

Since the sustainable development goals (SDGs) were established, various efforts have been developed globally to contribute to achieving the specific goals. In the field of health, specifically, there are reports of initiatives to contribute to SDG 3 focused on health, such as digital health [1, 2], health policies [3–6], and COVID-19 [7]. Because of the pandemic, many of the efforts may have served as previous experience to have a better response capacity, which is the case for countries with an excellent vaccination system [8–11]. Efforts to ensure sustainability during the pandemic are contained in Target 3.3: “By 2030, end the epidemics of AIDS, tuberculosis, malaria and neglected tropical diseases and combat hepatitis, waterborne diseases, and other communicable diseases.” On the other hand, target 3.4 is also currently an aspect that has been negatively impacted by COVID-19: mental health, which needs specific programs to treat people after the tremendous impact on their lives due to having witnessed diaries of deaths of friends and family, job losses, and impoverishment. Finally, the 3.8 targets are still pending since health coverage was the key to whether a person could live or die during the most challenging pandemic. After this pandemic, it is necessary to evaluate the remaining gaps to shorten them and provide the population with health coverage in general.

The implementation of social distancing [12, 13] and quarantines [14, 15] because of the COVID-19 pandemic [16] generated various mental health effects [17, 18]. Peru was severely damaged because both waves generated the country's highest mortality rate [19], which affected the population [20]. The population was exposed to fake news [21] and conspiracy theories [22], which generated technostress [23] and multitasking behavior [24]. The lack of adequate policies causes mental distress [25] and urgency for self-care behaviors [26], including the use of unproven drugs [27] and medicinal plants [28]. Previous research assessed the impact of social distancing measures during the early months of the COVID-19 pandemic in the United States and revealed that stay-at-home orders and social distancing policies significantly reduced infection and death rates. Notably, effectiveness varied across states, correlating with income, education, demographics, political affiliations, and media consumption. This information is crucial for contextualizing our study on military parents' mental health and vaccination intentions. The effectiveness of social distancing measures provides insights into potential variations in policy impact based on socioeconomic and demographic factors. In our study, akin to the challenges faced by Peru, we acknowledge the broader consequences of such policies on mental health, including exposure to misinformation and subsequent technostress.

A worldwide mass immunization campaign has been imperative to reduce cases and mortality [29]. Today, approximately 55.9% of the world's population has been immunized with at least two doses of the COVID-19 vaccine. In Peru, 70.5% of the population is vaccinated against COVID-19 [30]. Peru is the sixth-ranked country with the highest percentage of vaccination in Latin America [31]. However, to achieve success in ending the pandemic, a strategic vaccination scheme with broad coverage continues to be carried out judiciously and rapidly, evaluating the facilitating or limiting factors in the target population remaining to be immunized [32]. Worldwide, the vaccine acceptance rate against COVID-19 is 60%. In Europe, Denmark has the highest acceptance rate (80%) for vaccination; in Ecuador, the highest rate in Latin America was 97% [33]. In China, it was determined that 91.3% of the participants intended to vaccinate (ITV), and the influencing factors were gender, marital status, and risk perception [34]. On the other hand, in Peru, the national reality regarding vaccination intention is often regionally contrasted; the highest ITV is found in Lima province (81.4%), while the lowest rate was found in Madre de Dios (53.9%) and Ucayali (69.9%) [35]. Additionally, living in a rural area and the female gender were the main factors limiting the ITV, while the unstable economy, having clinical COVID-19, or being seriously ill with COVID-19 were linked with a higher ITV [35]. It is relevant to note that, according to the research by Bao and Huang [36], it was found that women are more responsive to social pressure, while men are more sensitive to financial incentives. These results, obtained in a different context, raise the possibility of gender differences in vaccine acceptability and intention to vaccinate, which could influence our findings in the study of military parents. Thus, considering these gender dynamics identified by Bao and Huang, it is essential to explore how such differences might impact vaccination intentions among military parents, especially in an environment where gender has already been identified as a critical factor in vaccination decision-making.

However, few studies on ITV children have been reported by parents. In a systematic review evaluating 44 studies and 317,055 parents from the USA, China, Saudi Arabia, India, European countries, and Oceanic countries, only 60.1% of these parents expressed a desired ITV for their children [37]. However, no study has yet been conducted in Peru on the impact of mental health on the ITV against COVID-19 in military personnel. There is evidence that affirms a probable positive impact of mental health (depression and anxiety) on the acceptability of the vaccine. However, no reports have been published describing the ITV of their children in military parents [38, 39]. As military personnel and health personnel have been on the front lines of organization and care for COVID-19 since the pandemic's beginning to safeguard national security, this remains variable to understand.

A similar investigation affirms that adults have a higher probability of ITV against COVID-19. However, only fear of the risk of having this disease was measured [40], but the influence of other psychological factors (anxiety, depression, posttraumatic stress, burnout syndrome, and mental health support), which could influence acceptability, were previously not considered and are analyzed in our study. Additional research on South Korean parents reported that age, higher education, family economy, and the number of children in the family unit positively impact parents' willingness to vaccinate their children. However, whether parental gender has statistical significance was not determined [41]. Likewise, other research excludes the male gender and has studied only maternal intentions [42]. Although our study includes a small proportion of male soldiers, it enables us to evaluate whether this variable influences the ITV of their children. Other studies in the United States have estimated parental vaccine acceptability at 44.2% and 61.9%. However, their findings are descriptive, and the effect of variables influencing ITV was not evaluated [43, 44]. In Peru, it was found that 75% of those surveyed had the ITV; however, the data were obtained through online questionnaires, which could lead to channeling and information bias. Contrary to this research, in which personal interviews were conducted with an official in charge of control, this study does not specify the acceptance intention, which may not be prudent for this specific region of northern Peru where COVID-19 has been very adversely hit during the pandemic [35].

Additionally, within our military parent study, this research allows us to delve into the potential influence of government policies and interventions on vaccination intentions and mental health outcomes. Drawing on the insights from Li et al.'s investigation into the effectiveness of nonpharmaceutical policies such as lockdowns and subsidies, we can examine how these measures might shape the attitudes towards vaccination and mental well-being among military parents. Li et al.'s findings, indicating that economic impacts are manageable and policies should be tailored to specific socioeconomic groups, provide a foundation for understanding how targeted interventions can be tailored for distinct demographic segments within the military parent community.

This study is poised to delve into a crucial and underexplored facet of public health, the COVID-19 vaccination intentions of military parents for their children. The significance of unraveling the intricacies within this demographic lies in safeguarding the health of military children and recognizing the unique challenges and stressors this population faces. Military families navigate distinct circumstances, including potential deployment-related stressors, frequent relocations, and the inherent demands of serving in the armed forces. In the global pandemic, understanding the vaccination intentions of military parents becomes paramount.

By explicitly addressing the factors associated with the COVID-19 vaccination intentions within this cohort, with a keen focus on mental health, we aim to shed light on the nuanced interplay between psychological well-being and vaccine decision-making. This study is not merely an exploration of statistical associations; instead, it is a strategic effort to bridge the gap between the mental health challenges experienced by military parents and their decisions regarding the health and well-being of their children.

The importance of this research extends beyond the immediate scope of COVID-19, as insights gained can inform targeted interventions and policies tailored to the unique needs of military families. As frontline defenders, their health decisions bear broader implications for the overall readiness and resilience of the armed forces. Thus, unraveling the intricate web of mental health and associated factors influencing vaccination intentions, we contribute valuable knowledge that can shape public health strategies and support systems for military families, fostering a comprehensive and tailored approach to vaccination efforts.

Therefore, the aim was to establish the prevalence and factors associated with ITV children against COVID-19 in military parents in Lambayeque, Peru, 2021.

## 2. Materials and Methods

### 2.1. Study Design

It was an analytical cross-sectional study of secondary data analysis using the database of a primary study to evaluate factors linked with mental health illness in military personnel in the Lambayeque region in the second wave of the COVID-19 pandemic in November 2021 [45]. The specific data collection from only one city can limit the generalization of the outcomes. Also, the lack of control of confounding variables could influence vaccination intentions; therefore, this is a limitation that is recognized in the current study.

### 2.2. Space and Time

The preliminary study was conducted from November 02 to 09, 2021, during the second COVID-19 pandemic in the Lambayeque-Peru region. Lambayeque has been the tenth most afflicted region in the COVID-19 pandemic [46], with 104,403 cases by February 20, 2022.

### 2.3. Sampling

The study population was military personnel in a health emergency area in November 2021 in the Lambayeque Peru Region. It used a nonprobabilistic convenience sampling. The list of the VII Infantry Brigade (Lambayeque, Cajamarca, and Ancash) was requested. In the primary study, we estimated a population size of 820 military personnel. Expecting a prevalence of 12.8% based on relevant literature, we aimed for a robust statistical foundation with a 99% confidence level and a precision of 2.5%. This calculation resulted in an initial sample size requirement of 485 individuals. A prudent approach was adopted, incorporating a 10% rejection rate and a 10% allowance for incomplete registrations to account for potential rejection and incomplete registrations. Consequently, the adjusted required sample size for our study was 582 individuals. During the data collection phase, we successfully enrolled 710 military personnel who willingly participated in the study. Nevertheless, 95 military personnel were not included in the final analysis as their responses on the Patient Health Questionnaire 9 (PHQ-9) instrument (depression) and General Anxiety Disorder-7 (GAD-7) instrument (anxiety) were incomplete. Consequently, the final sample size for the primary study was 615 military personnel. From this secondary study, 201 individuals reported having children and were specifically selected for inclusion in this analysis.

We estimated statistical power considering covariates such as suicidal risk, personal mental history, and Catholic religion. For suicidal risk, a statistical power of 53.03% was obtained, utilizing vaccination intention proportions in parents without suicidal risk (*p*1=0.927) and with suicidal risk (*p*2=0.750), along with the group without suicidal risk (*n*1 = 124) and with suicidal risk (*n*2 = 12). Regarding the personal mental history factor, we employed vaccination intention proportions in parents without a mental history (*p*1=0.935) and with a mental history (*p*2=0.500), as well as the group without a mental history (*n*1 = 199) and with a mental history (*n*2 = 2), resulting in a statistical power of 60.36%. Finally, for the variable “Catholic,” a statistical power of 82.68% was estimated, considering vaccination intention proportions in non-Catholics (*p*1=0.800) and Catholics (*p*2=0.958), along with the group of non-Catholic parents (*n*1 = 35) and Catholic parents (*n*2 = 166). The sample size, the magnitude of the expected effect, the level of statistical significance, the variability of the data, and the validity of the assumptions made have been considered to ensure the power of the analysis.

### 2.4. Selection Criteria

All military personnel on duty for at least one month during the COVID-19 health emergency in Lambayeque in 2021 were included in the study. All military personnel who had requested their temporary leave due to COVID-19 risk factors, performed remote work in home isolation after testing positive for COVID-19, and belonged to other regions of Peru were excluded from the study. The criterion for elimination was the inadequate completion of the data collection form.

### 2.5. Variables and Instruments

The dependent variable was the ITV children reported by military parents. The independent mental health variables were history of mental health, seeking mental health support, resilience, anxiety, depression, burnout, posttraumatic stress, and suicidal risk. The instruments used are described as follows:

- Abbreviated Connor–Davidson Scale (CD-RISC): Ten items that measure resilience globally through a Likert scale with a score of 0–4, whose relationship between the score and the presence of resilience is directly proportional and is classified according to quartiles (low: first quartile, middle: second and third quartiles, and high: fourth quartile) [47]. It has been validated in older adults [48], health personnel [49], young adults [50], and Spanish-speaking fibromyalgia patients [51].
- Generalized anxiety disorder 7-item scale (GAD-7): Seven questions and assesses the frequency of symptoms during the last 2 weeks [52]. It was validated among a group of immigrants of Hispanic origin living in the USA [53]. A cutoff point of 10 or more represents the optimal compromise between sensitivity and specificity (>80%) [52–54] and is associated with a positive predictive value of >99% and a negative predictive value of 0.833 [54].
- Patient Health Questionnaire-9 (PHQ-9): It is a self-administered version measured according to a Likert scale consisting of nine criteria to evaluate significant depression [55] whose cutoff point greater than and equal to 10 represents an optimal compromise between sensitivity and high specificity [56]. It was validated by analyzing secondary data from the 2016 Encuesta Demográfica y de Salud Familiar (2016 ENDES) (30,449 Peruvians), which reported consistently good measurement invariance and optimal reliability [57].
- Maslach Burnout Inventory Human Services Survey (MBI-HSS): Considering the gold standard for measuring burnout syndrome, it consists of 22 items and 3 domains: emotional exhaustion, depersonalization, and personal fulfillment at work and has been validated in Chilean and Peruvian health workers [58, 59]. Cutoff points: EB > 26, and DP > 9, PF < 34, where EB = emotional burnout, PF = personal fulfillment, and DP = depersonalization [59].
- Posttraumatic stress disorder (PTSD) checklist (PCL-C): Consisting of 17 questions, it explores the re-experiencing of the trauma, trauma avoidance blunting, and hyperarousal symptoms and defines PTSD as a score of 43 or more [60]. It has been validated in the Chilean population after the 2010 earthquake in Chile [61] and used to evaluate PTSD in Peruvians after the 2007 earthquake in Pisco, Peru, presenting excellent psychometric properties [62].
- Plutchik's Suicide Risk Scale is a self-administered questionnaire of 15 questions, with yes/no answers. Each yes scores 1. A score of 6 or more indicates the presence of suicidal risk [63]. It has been validated in a prison sample of Mexican youths, proposing a percentile cutoff point (no risk: <25, high risk: >80) [64], and in Colombian adolescents, the items with the highest factor load were suicidal ideation and attempt [65].

### 2.6. Data Collection, Management, and Analysis

Face-to-face interviews were conducted using structured questionnaires to collect sociodemographic information, variables related to physical and mental health, as well as scales to assess resilience and suicide risk, depression, posttraumatic stress disorder (PTSD), anxiety, and burnout syndrome. These interviews were conducted in three groups and two shifts (morning and afternoon) for two hours and under the supervision of an officer responsible for control. The REDCap system collected and validated the information entered to ensure data quality. The dependent variable of intention to vaccinate children was evaluated with the following question: “Would you vaccinate your child against COVID-19? Stata v.17.0 was then used for statistical analysis.” We analyzed the intention to vaccinate as a binomial outcome. Sociodemographic polytomous categorical variables included age, sex, marital status, educational level, and religion; childbearing was dichotomous. Body mass index and working time were continuous numerical variables. Physical health variables included alcohol and tobacco use, history of hypertension, and diabetes; they were analyzed as dichotomous. Mental health variables were seeking mental health support and a history of personal or family mental health disorders. Likewise, food insecurity and trust in government were dichotomous categorical variables. Univariate analysis of the variables was performed, obtaining means and standard deviations for quantitative variables with normal distribution; median and interquartile ranges were reported, and frequencies and proportions for qualitative variables were reported.

In the bivariate analysis, different tests were used to compare proportions and numerical variables. Students' *t*-tests were used for numerical variables, while the chi-square test was used for proportions. For those variables without normal distribution, the Mann–Whitney *U* test. The significance level used was 5%. The simple and multiple regression analysis estimated factors associated with intention to vaccinate using the prevalence ratio (PR) and 95% confidence intervals (CI 95%). Also, it used generalized linear models (GLM) with Poisson distribution, log-link function, and robust variance.

### 2.7. Ethical Considerations and Dissemination

The confidentiality of each study participant was respected by using a code and avoiding using their names. The data collected were handled solely and exclusively by the research team and in a data storage platform that did not include identifying variables of the study participants. The Ethics Committee approved oficio No's study. 269-2021-CIEI-FMH-USMP from Universidad de San Martín de Porres.

## 3. Results

### 3.1. Psychosocial and Occupational Characteristics of Military Parents

Of the 201 military personnel evaluated, the majority were male (92.5%), and the median age was 40. 82.6% were of the Catholic faith and 27.4% reported frequent alcohol consumption. More than half of the military personnel had 19 months or more working on the front line before COVID-19 (55.6%). Nine percent reported seeking mental health help during the COVID-19 pandemic. It was reported anxiety (20.3%), depression (6.5%), and posttraumatic stress disorder (6.5%). Most reported ITV in their children against COVID-19 (93%) (Table 1).

### 3.2. Mental Health and Other Factors Associated with COVID-19 Vaccination Intention toward Children of Military Parents in Bivariate Analysis

In Table 2, the factors linked with ITV were marital status (*p*=0.001), Catholic faith (*p*=0.002), personal history of mental health (*p*=0.016), military work time (*p*=0.010), and suicidal risk (*p*=0.039).

### 3.3. Mental Health and Other Factors Associated with COVID-19 Vaccination Intention toward Children of Military Parents in Simple and Multiple Regression Analysis

In the multiple models, the association was maintained for only two variables (religious faith and mental health help-seeking). In the simple model, it was determined that being divorced (PR = 1.21; 95% CI: 1.04–1.40), being of the Catholic faith (PR = 1.20; 95% CI: 1.01–1.42), having a family history of mental illness (PR = 1.08; 95% CI: 1.04-1.12), having sought mental help during the pandemic (PR = 1.08; 95% CI: 1.04–1.13), and having burnout syndrome (PR = 1.09; 95% CI: 1.04–1.15) were associated with a higher prevalence of intention to vaccinate in children. Catholics had a 23% higher prevalence of intention to vaccinate their children (PR = 1.23; 95% CI: 1.01–1.50). Having sought mental help increased the prevalence of intention to vaccinate by 8% (PR = 1.08; 95% CI: 1.00–1.15) (Table 3).

## 4. Discussion

### 4.1. Prevalence of Vaccination Intention toward Children of Military Parents

It was determined that 9 out of 10 military personnel reported intending to vaccinate their minor children against COVID-19, which is similar to the findings reported by Urrunaga-Pastor et al. in Latin America and by Zhang et al. in China, where 90% and 93.4% of parents accepted vaccination of their children or adolescents against COVID-19 [39, 66]. It has also been reported that Peru has the lowest reluctance to vaccinate (7.3%) [39]. Likewise, a study conducted in Brazil estimated a reluctance to vaccinate against COVID-19 of 2.8%, even in parents who were reluctant to use other vaccines [67]. However, a study conducted in Japan reported that only 64.7% of parents would vaccinate their children [68], and other studies conducted in the USA, Switzerland, and Latin America reported 67% [69], 59.2% [70], and 69.2% acceptance [71], respectively. In addition, our study found lower vaccination intention rates than those observed in other regions. In Korea, parents' average vaccination intention score for their children was 2.96 out of 7 points. In Egypt, 40.1% of parents expressed an intention to vaccinate their children against COVID-19 if available. In Oman, 56% of mothers with children aged 5 to 11 reported a likelihood or substantial likelihood of accepting vaccination. In Israel, 57% of parents with children aged 5 to 11 expressed an intention to vaccinate their children against COVID-19. However, these data are not comparable to this Peruvian study as, at the time of their execution, vaccines for minor children were not a real possibility, and clinical trials had not yet been completed in their entirety, generating greater distrust in the safety and efficacy of the vaccines.

The high intention to vaccinate is explained by an increased concern for the well-being of children, who have been negatively affected in their growth, development, and mental health, secondary to school closures, family deaths, and higher rates of domestic violence during COVID-19 confinement [72, 73]. Similarly, job loss, decreased income, and fear of COVID-19 infection contributed to parental stress, and parents perceived the vaccine as a mitigation strategy for this complex disease [74].

### 4.2. Mental Health and Other Factors Associated with COVID-19 Vaccination Intention toward Children of Military Parents

Seeking mental health support during the state of emergency due to COVID-19 increases the prevalence of vaccination intention in children by 8%, which is similar to the outcome from Murphy et al. when analyzing an Irish study, where they found that those who doubted the vaccine were less likely to have received treatment for a mental health problem (odds ratio = 0.63), compared to those who accepted it [75]. Likewise, a study conducted in Latin America reported that those participants who had symptoms of anxiety (PR = 0.87) and depressive symptoms (PR = 0.93) were associated with higher parental intention to vaccinate their children and adolescents against COVID-19 [39]. This finding could be justified because parents with such clinical conditions value the usefulness of vaccinations to a greater extent, as they are a vulnerable group to this disease [76]. However, Nguyen et al. found that concerns about vaccination and lack of trust in the government, doubt about side effects, and efficacy of vaccines were higher among parents with any symptoms of anxiety or depression compared with those without symptoms. In addition, they reported that participants with mental health disorders had more significant barriers to accessing a vaccine, especially women [77], which could be explained by the fact that they spend more time caring for children and the more remarkable inability to take time off work to get vaccinated. One potential explanation for this association is the heightened awareness and health consciousness often accompanying individuals seeking mental health support. Military parents experiencing mental health challenges during the pandemic may develop a heightened sensitivity to health-related concerns, recognizing their vulnerability to severe outcomes from COVID-19. This increased awareness could lead to a proactive approach to health-promoting behaviors, including a more positive attitude toward vaccination for their children. From a psychosocial perspective, engaging with mental health support services may empower parents to prioritize holistic health for their families. Seeking mental health support could serve as a catalyst for adopting preventive health measures and viewing vaccination as a crucial component of safeguarding their children's well-being. The act of seeking help itself may contribute to a sense of responsibility and commitment to protective health behaviors, influencing the decision-making process regarding vaccination.

Having burnout syndrome was positively associated with a higher prevalence of intention to vaccinate in children in the crude model; however, no significant differences were found in the adjusted model. Preexisting burnout could explain this finding in burnout syndrome exacerbates the sense of danger and symptoms of distress about the likelihood of becoming ill or dying from COVID-19 [78]. Thus, the fear that their children will have adverse outcomes from infection translates into an increased intention to vaccinate. There are no studies directly assessing burnout syndrome and vaccination intention, but Yigit et al. [74] presented a similar finding, reporting that parents with high anxiety levels regarding COVID-19 infection had higher vaccine acceptability for themselves and their children. However, these findings differ from those of Temsah et al. [79], where those parents with higher anxiety levels about possible adverse side effects of the vaccine were less likely to vaccinate their children.

Having a family member with mental health problems was positively associated with a higher prevalence of vaccination intention in children in the crude model; however, no significant differences were found in the adjusted model. However, Milan et al. found that families whose mothers had a previous diagnosis of PTSD had significantly lower confidence in the COVID-19 vaccine and less intention to vaccinate themselves or their children. These findings were explained by greater institutional distrust, less scientific belief, and less interest in basing their vaccine decisions on government and healthcare sources [80]. The positive association between having a family member with mental health problems and a higher prevalence of vaccination intention in children in the crude model may be explained by an increased awareness of health risks and a heightened sense of vulnerability in these families. Individuals experiencing mental health issues or having close family members with such problems might be more attuned to health-related threats, including the risks posed by infectious diseases such as COVID-19. This heightened awareness could lead to a greater willingness to vaccinate children as a preventive measure. Additionally, the shared experience of dealing with mental health challenges within the family might foster a collective approach to health protection, influencing vaccination decisions.

It was found that having the Catholic faith increased the prevalence of ITV in children by 23%, which is similar to that reported by Corcoran et al., where it was observed that nonevangelical Protestants, Catholics, Jews, and atheists were more likely to have received a COVID-19 vaccine compared to evangelical Protestants (OR 2.02) [81]. The Catholic Church has supported the vaccination program of different governments and has allowed their Church facilities to be community vaccination centers [82]. However, Williams et al. found no statistical significance between parental reluctance to vaccinations and adherence to major religious traditions in a group of 250 Latino Christian caregivers in the USA [83]. Our finding differs from that reported in South Korea, where 298 parents of children aged 5 to 11 participated, and religion did not emerge as a significantly associated factor with vaccination intention. It also differs from a multicentric study conducted in 90 countries, which found that Christianity was associated with a lower rate of COVID-19 vaccination. These findings show that faith-based religious resistance to vaccination is rare and that if it exists, it is mediated by distrust of science and political affiliations rather than theology [84]. One potential mechanism underlying this association could be the strong influence of religious beliefs on health-related decision-making within the Catholic community. Catholic teachings often emphasize the value of life and the importance of safeguarding the well-being of individuals, aligning with the broader public health goals of vaccination. The moral and ethical dimensions associated with the Catholic faith might amplify the perceived importance of protecting children from the severe consequences of COVID-19, thus fostering a higher intention to vaccinate. Additionally, the sense of community and shared values within religious groups can play a role in shaping health behaviors. In the context of vaccination, the Catholic faith community may provide a supportive environment where vaccine-related information is disseminated and positive attitudes toward vaccination are reinforced. Social norms within religious communities can influence individual attitudes, potentially contributing to the observed increased prevalence of COVID-19 vaccination intention in children among military parents of the Catholic faith.

### 4.3. Implication of Findings in Public Health and Mental Health

The high prevalence of intention to vaccinate observed in this demographic, notably 9 out of 10 military personnel expressing their commitment to vaccinating their minor children against COVID-19, underscores the importance of targeted vaccination campaigns within military communities. Understanding the driving forces behind this intention, such as a heightened concern for children's well-being amidst the challenges posed by the pandemic, underscores the need for tailored public health messaging. As the military plays a crucial role in national defense, ensuring the health and resilience of military families through effective vaccination initiatives safeguards individual well-being and contributes to broader national health security.

The salient findings revealing the significant associations between mental health factors (such as previously sought mental health assistance, burnout, and mental health history in the family), Catholic faith, and vaccination intention among military parents carry profound implications for public health strategies within the military context. Addressing mental health concerns is pivotal, as the positive association between vaccination intention and seeking mental health assistance highlights the interconnectedness of mental well-being and preventive health behaviors. Implementing targeted mental health support programs tailored to the unique challenges faced by military parents can foster a healthier community and positively influence vaccination decision-making. The impact of religious beliefs, mainly Catholic faith, on vaccination intention, underscores the importance of respecting and accommodating diverse religious perspectives within the military population. Crafting vaccine communication strategies that align with religious values can enhance vaccine acceptance and contribute to building trust in the military healthcare system. Furthermore, recognizing the influence of burnout and a family history of mental health issues emphasizes the need for holistic support mechanisms. Implementing interventions to alleviate burnout and providing resources for families with mental health histories can contribute to the overall resilience and well-being of military families.

### 4.4. Limitations

This research has some limitations. Firstly, its methodology does not allow the ability to attribute causality between mental health and intention to vaccinate against COVID-19. Secondly, the potential selection bias because the prevalence of vaccination does not represent the prevalence of the general national population since the preliminary study included only one regional site in the Northern Region of Peru. Additionally, there could be an information bias since it was impossible to measure the educational level, political party affiliation, economic security, food security [35], parental beliefs, financial constraints during the COVID period, the number of children, parental COVID-19 vaccination history, experience of side effects and postvaccination symptoms in parents, parental history of COVID, the extent to which parents perceived pressure from their surroundings to vaccinate their children against COVID-19, perceived behavioral control over COVID-19 vaccination, and attitudes toward COVID-19 vaccination, which have been shown to influence vaccination intention. Despite these limitations, the outcomes have relevant implications for public and mental health professionals in designing psychological interventions in the military population, which is exposed to high workloads and stress exacerbated by the pandemic. Likewise, this is the first study that evaluates the association between mental health and vaccination intention in the Peruvian military for their children. The specific data collection from only one city can limit the generalization of the outcomes. Also, the lack of control of confounding variables could influence vaccination intentions; therefore, this is a limitation that is recognized in the current study. Due to this, participants may have overreported their vaccination intentions or underreported mental health concerns.

### 4.5. Recommendations

The results of this study highlight the usefulness of implementing strategies focused on populations vulnerable to worse outcomes by COVID-19, such as patients with mental health disorders [85]. Considering the increase in the number of people living with mental health disorders after the pandemic, coupled with the neglect and stigma that still prevail in Peruvian society, there is an imminent need to address mental health in the population through more specialized primary health care [86]. Vaccination against COVID-19 is positively related to improving mental health in the population; therefore, health professionals and government authorities should work together to pay significantly greater attention to this group of patients and ensure their ultimate immunization.

Recommendations for future research include carrying out longitudinal studies to better understand the causality between mental health factors and vaccination intentions. Future research could adopt a longitudinal design. Therefore, broader sampling strategies are needed to expand the study to include a wider range of military personnel across different regions, which could enhance the generalizability of the findings. Also, it is relevant to conduct qualitative insights, as incorporating qualitative methods could provide deeper insights into the personal experiences and beliefs that underlie vaccination intentions.

## 5. Conclusions

Finding mental health support, experiencing burnout syndrome, having a relative who suffers from mental health problems, and being part of the Catholic religion were associated with a higher willingness to immunize the children of Peruvian military members. The study opens avenues for further research to explore the complex interplay between mental health, religious beliefs, and vaccination intentions in different populations and settings.

## Figures and Tables

**Table 1:** Lambayeque military characteristics in November 2021.

Characteristics	*N* (%)
Age (years)^*∗*^	40 (32–44)
Gender	
Female	15 (7.5)
Male	185 (92.5)
Marital status	
Single	35 (17.4)
Married	148 (73.6)
Cohabitant	11 (5.5)
Divorced	7 (3.5)
Catholic faith	
No	35 (17.4)
Yes	166 (82.6)
Alcoholism	
No	146 (72.6)
Yes	55 (27.4)
Hypertension	
No	185 (92.0)
Yes	16 (8.0)
Diabetes	
No	191 (95.0)
Yes	10 (5.0)
Mental health history (personal)	
No	199 (99.0)
Yes	2 (1.0)
Mental health history (familiar)	
No	193 (96.5)
Yes	7 (3.5)
Previously sought mental health assistance	
No	183 (91.0)
Si	18 (9.0)
Trust in the government to manage COVID-19	
Yes	107 (53.2)
No	94 (46.7)
Military employment time	
1 to 6 months	15 (8.3)
7 to 12 months	22 (12.2)
8 to 18 months	43 (23.9)
19 months or more	100 (55.6)
Food insecurity	
No	101 (50.3)
Yes	100 (49.8)
Resilience	
Low	92 (57.1)
High	69 (42.9)
Anxiety	
No	130 (79.8)
Yes	33 (20.3)
Depression	
No	158 (93.5)
Yes	11 (6.5)
Burnout syndrome	
No	144 (90.6)
Yes	15 (9.4)
Posttraumatic stress disorder	
No	158 (93.5)
Yes	11 (6.5)
Suicide risk	
No	124 (91.2)
Yes	12 (8.8)
Intention to vaccinate children	
No	14 (7.0)
Yes	187 (93.0)

^*∗*^Median (25th percentile–75th percentile).

**Table 2:** Characteristics linked with ITV (November 2021).

Variables	Intention to vaccinate		*p* value^*∗*^
	No (*n* = 14) *n* (%)	Yes (*n* = 187) *n* (%)	
Age (years)	33 (21–44)^*∗∗*^	40 (32–44)^*∗∗*^	0.060^*∗∗∗*^
Gender			0.958
Female	1 (6.7)	14 (93.3)	
Male	13 (7.0)	172 (93.0)	
Marital status			**0.001**
Single	6 (17.1)	29 (82.9)	
Married	5 (3.4)	143 (96.6)	
Cohabitant	3 (27.3)	8 (72.7)	
Divorced	0 (0.0)	7 (100.0)	
Catholic faith			**0.002**
No	7 (20.0)	28 (80.0)	
Yes	7 (4.2)	159 (95.8)	
Alcoholism			0.916
No	10 (6.9)	136 (93.2)	
Yes	4 (7.3)	51 (92.7)	
Hypertension			0.054
No	11 (6.0)	174 (94.1)	
Yes	3 (18.8)	13 (81.3)	
Diabetes			0.699
No	13 (6.8)	178 (93.2)	
Yes	1 (10.0)	9 (90.0)	
Mental health history (personal)			**0.016**
No	13 (6.5)	186 (93.5)	
Yes	1 (50.0)	1 (50.0)	
Mental health history (familiar)			0.460
No	14 (7.3)	179 (92.8)	
Yes	0 (0.0)	6 (100.0)	
Have previously sought mental health assistance			0.224
No	14 (7.7)	169 (92.4)	
Yes	0 (0.0)	18 (100.0)	
Trust in the government to manage COVID-19			0.761
Yes	8 (7.5)	99 (92.5)	
No	6 (6.4)	88 (93.6)	
Military employment working time			**0.010**
1 to 6 months	4 (26.7)	11 (73.3)	
7 to 12 months	2 (9.1)	20 (90.9)	
8 to 18 months	2 (4.7)	41 (95.4)	
19 months or more	4 (4.0)	96 (96.0)	
Food insecurity			0.259
No	5 (5.0)	96 (95.1)	
Yes	9 (9.0)	91 (91.0)	
Resilience			0.488
Low	8 (8.7)	84 (91.3)	
High	4 (5.8)	65 (94.2)	
Anxiety			0.241
No	8 (6.2)	122 (93.9)	
Yes	4 (12.1)	29 (87.9)	
Depression			0.980
No	9 (7.3)	115 (92.7)	
Yes	3 (7.1)	39 (92.9)	
Burnout syndrome			0.245
No	12 (8.3)	132 (91.7)	
Yes	0 (0.0)	15 (100.0)	
Posttraumatic stress disorder			0.790
No	11 (7.0)	147 (93.0)	
Yes	1 (9.1)	10 (90.9)	
Suicide risk			**0.039**
No	9 (7.3)	115 (92.7)	
Yes	3 (25.0)	9 (75.0)	

^*∗*^ *p* value calculated using the chi-square test (categorical variables). ^*∗∗*^*p* value calculated using the *U* test (Mann–Whitney) (categorical-numerical variables). ^*∗∗∗*^Median-interquartile range. The bold values mean that these values are statistically significant.

**Table 3:** Factors linked with IFV in simple and multiple regression analysis.

Characteristics	Intention to vaccinate children					
	Simple regression			Multiple regression		
	PR	IC 95%	*p* value^*∗*^	PR	IC 95%	*p* value^*∗*^
Age (years)	1.01	1.00–1.01	**0.049**	1.00	0.49–0.97	0.791
Gender						
Female	Ref.					
Male	1.00	0.86–1.15	0.957			
Marital status						
Single	Ref.			Ref.		
Married	1.17	0.99–1.36	0.051	1.12	0.93–1.36	0.225
Cohabitant	0.88	0.59–1.30	0.515	0.76	0.46–1.25	0.280
Divorced	1.21	1.04–1.40	**0.015**	1.13	0.94–1.36	0.196
Catholic faith						
No	Ref.			Ref.		
Yes	1.20	1.01–1.42	**0.037**	1.23	1.01–1.50	**0.049**
Alcoholism						
No	Ref.					
Yes	1.00	0.91–1.09	0.918			
Hypertension						
No	Ref.					
Yes	0.86	0.68–1.10	0.230			
Diabetes						
No	Ref.					
Yes	0.97	0.78–1.19	0.746			
Mental health history (personal)						
No	Ref.					
Yes	0.53	0.13–2.15	0.378			
Mental health history (familiar)						
No	Ref.			Ref.		
Yes	1.08	1.04–1.12	**<0.001**	1.04	0.94–1.16	0.468
Previously sought mental health assistance						
No	Ref.			Ref.		
Yes	1.08	1.04–1.13	**<0.001**	1.08	1.00–1.15	**0.037**
Trust in the government to manage COVID-19						
Yes	Ref.					
No	1.01	0.94–1.09	0.761			
Military working time						
1 to 6 months	Ref.					
7 to 12 months	1.24	0.89–1.73	0.207			
8 to 18 months	1.30	0.95–1.78	0.100			
19 months or more	1.31	0.96–1.78	0.087			
Food insecurity						
No	Ref.					
Yes	0.96	0.89–1.03	0.263			
Resilience						
Low	Ref.					
High	1.03	0.95–1.12	0.478			
Anxiety						
No	Ref.					
Yes	0.94	0.82–1.07	0.339			
Depression						
No	Ref.					
Yes	1.00	0.91–1.10	0.980			
Burnout syndrome						
No	Ref.			Ref.		
Yes	1.09	1.04–1.15	**0.001**	1.03	0.99–1.07	0.183
Posttraumatic stress disorder						
No	Ref.					
Yes	0.98	0.81–1.18	0.813			
Suicide risk						
No	Ref.					
Yes	0.81	0.58–1.13	0.209			

^*∗*^ *p* values obtained using generalized linear models (GLM), *Poisson* family, log-link function, robust variance. The bold values mean that these values are statistically significant.

## Data Availability

The data supporting this study's findings are available on request from the authors.

## References

[1] Asi Y. M., Williams C. (2018). The role of digital health in making progress toward Sustainable Development Goal (SDG) 3 in conflict-affected populations. *International Journal of Medical Informatics*.

[2] Wyllie J., Carlson J., Heinsch M., Kay-Lambkin F., McCoy A. (2022). eHealth services and SDG3: increasing the capacity of care. *Australasian Marketing Journal*.

[3] Eckermann E. (2018). Sdg 3: a missed opportunity to transform understandings and monitoring of health, well-being and development?. *Applied Research in Quality of Life*.

[4] Bjegovic-Mikanovic V., Abousbie Z. A. S., Breckenkamp J. (2019). A gap analysis of SDG 3 and MDG 4/5mortality health targets in the six Arabic countries of North Africa: Egypt, Libya, Tunisia, Algeria, Morocco, and Mauritania. *Libyan Journal of Medicine*.

[5] Olaniyi P. O., Oluwatobi F. T., Olaniyan M. E. (2022). Healthcare systems strengthening in Africa: the call for action to achieve SDG 3. *The International Journal of Health Planning and Management*.

[6] Yang S.-Y., Liu C., Hsieh P. L. (2022). Effects of team-based learning on students’ teamwork learning attitude, and health care competence for older people in the community to achieve SDG-3. *International Journal of Environmental Research and Public Health*.

[7] van Zanten J. A., van Tulder R. (2020). Beyond COVID-19: applying “SDG logics” for resilient transformations. *Journal of International Business Policy*.

[8] Shaw J., Stewart T., Anderson K. B. (2021). Assessment of US healthcare personnel attitudes towards coronavirus disease 2019 (COVID-19) vaccination in a large university healthcare system. *Clinical Infectious Diseases*.

[9] Drissi Bourhanbour A., Ouchetto O. (2021). Morocco achieves the highest COVID-19 vaccine rates in Africa in the first phase: what are reasons for its success?. *Journal of Travel Medicine*.

[10] Ezeude G. I., Glover K. R., Nieves Santiago A. A., Repella E., Tang E. (2022). Roadblocks and successes in preparing COVID-19 vaccination clinics: perspectives from pharmacy residents. *American Journal of Health-System Pharmacy*.

[11] Walkowiak M. P., Walkowiak D. (2021). Predictors of COVID-19 vaccination campaign success: lessons learnt from the pandemic so far. A case study from Poland. *Vaccines*.

[12] Sun C., Zhai Z. (2020). The efficacy of social distance and ventilation effectiveness in preventing COVID-19 transmission. *Sustainable Cities and Society*.

[13] Olivera-La Rosa A., Chuquichambi E. G., Ingram G. P. D. (2020). Keep your (social) distance: pathogen concerns and social perception in the time of COVID-19. *Personality and Individual Differences*.

[14] Pietrabissa G., Simpson S. G. (2020). Psychological consequences of social isolation during COVID-19 outbreak. *Frontiers in Psychology*.

[15] Hamza C. A., Ewing L., Heath N. L., Goldstein A. L. (2021). When social isolation is nothing new: a longitudinal study on psychological distress during COVID-19 among university students with and without preexisting mental health concerns. *Canadian Psychology/Psychologie canadienne*.

[16] World Health Organization (2022). *Novel Coronavirus (2019-Ncov) Report No: Situation Report-1*.

[17] Kim A. W., Nyengerai T., Mendenhall E. (2022). Evaluating the mental health impacts of the COVID-19 pandemic: perceived risk of COVID-19 infection and childhood trauma predict adult depressive symptoms in urban South Africa. *Psychological Medicine*.

[18] Wilbiks J. M. P., Best L. A., Law M. A., Roach S. P. (2021). Evaluating the mental health and well-being of Canadian healthcare workers during the COVID-19 outbreak. *Healthcare Management Forum*.

[19] Echeverría Ibazeta R. R., Sueyoshi Hernandez J. H. (2020). Epidemiological situation of COVID-19 in South America. *Revista de la Facultad de Medicina Humana*.

[20] León-Jiménez F., Vives-Kufoy C., Failoc-Rojas V. E., Valladares-Garrido M. J. (2021). Mortalidad en pacientes hospitalizados por COVID-19. Estudio prospectivo en el norte del Perú, 2020. *Revista Medica de Chile*.

[21] Balakrishnan V., Ng W. Z., Soo M. C., Han G. J., Lee C. J. (2022). Infodemic and fake news – a comprehensive overview of its global magnitude during the COVID-19 pandemic in 2021: a scoping review. *International Journal of Disaster Risk Reduction*.

[22] Pummerer L., Böhm R., Lilleholt L., Winter K., Zettler I., Sassenberg K. (2022). Conspiracy theories and their societal effects during the COVID-19 pandemic. *Social Psychological and Personality Science*.

[23] Camacho S., Barrios A. (2022). Teleworking and technostress: early consequences of a COVID-19 lockdown. *Cognition, Technology and Work*.

[24] Qin X., Clemmensen T., Xin H. (2023). How motivation, nomophobic design and environmental demands predict students’ media multitasking when participating in online courses during COVID-19: an empirical study with a HCI time and temporality lens. *Interacting with Computers*.

[25] Guttormson J. L., Calkins K., McAndrew N., Fitzgerald J., Losurdo H., Loonsfoot D. (2022). Critical care nurse burnout, moral distress, and mental health during the COVID-19 pandemic: a United States survey. *Heart and Lung*.

[26] Petrova D., Salamanca-Fernández E., Rodríguez Barranco M., Navarro Pérez P., Jiménez Moleón J. J., Sánchez M.-J. (2020). La obesidad como factor de riesgo en personas con COVID-19: posibles mecanismos e implicaciones. *Atención Primaria*.

[27] Shrestha A. B., Aryal M., Magar J. R., Shrestha S., Hossainy L., Rimti F. H. (2022). The scenario of self-medication practices during the covid-19 pandemic; a systematic review. *Annals of medicine and surgery (2012)*.

[28] Al-kuraishy H. M., Al-Fakhrany O. M., Elekhnawy E. (2022). Traditional herbs against COVID-19: back to old weapons to combat the new pandemic. *European Journal of Medical Research*.

[29] Lurie N., Saville M., Hatchett R., Halton J. (2020). Developing covid-19 vaccines at pandemic speed. *New England Journal of Medicine*.

[30] Mathieu E., Ritchie H., Ortiz-Ospina E. (2021). A global database of COVID-19 vaccinations. *Nature Human Behaviour*.

[31] Who (2022). WHO coronavirus (COVID-19) dashboard. https://covid19.who.int.

[32] Ezati Rad R., Kahnouji K., Mohseni S. (2022). Predicting the COVID-19 vaccine receive intention based on the theory of reasoned action in the south of Iran. *BMC Public Health*.

[33] Sallam M. (2021). COVID-19 vaccine hesitancy worldwide: a concise systematic review of vaccine acceptance rates. *Vaccines*.

[34] Wang J., Jing R., Lai X. (2020). Acceptance of COVID-19 vaccination during the COVID-19 pandemic in China. *Vaccines*.

[35] Herrera-Añazco P., Uyen-Cateriano Á, Urrunaga-Pastor D. (2021). Prevalencia y factores asociados a la intención de vacunarse contra la COVID-19 en el Perú. *Revista Peruana de Medicina Experimental y Salud Pública*.

[36] Bao Z., Huang D. (2020). *Gender Differences in Reactions to Enforcement Mechanisms: A Large-Scale Natural Field Experiment*.

[37] Galanis P., Vraka I., Siskou O., Konstantakopoulou O., Katsiroumpa A., Kaitelidou D. (2022). Willingness, refusal and influential factors of parents to vaccinate their children against the COVID-19: a systematic review and meta-analysis. *Preventive Medicine*.

[38] Bendau A., Plag J., Petzold M. B., Ströhle A. (2021). COVID-19 vaccine hesitancy and related fears and anxiety. *International Immunopharmacology*.

[39] Urrunaga-Pastor D., Herrera-Añazco P., Uyen-Cateriano A. (2021). Prevalence and factors associated with parents’ non-intention to vaccinate their children and adolescents against COVID-19 in Latin America and the caribbean. *Vaccines*.

[40] Sherman S. M., Smith L. E., Sim J. (2021). COVID-19 vaccination intention in the UK: results from the COVID-19 vaccination acceptability study (CoVAccS), a nationally representative cross-sectional survey. *Human Vaccines and Immunotherapeutics*.

[41] Choi S.-H., Jo Y. H., Jo K. J., Park S. E. (2021). Pediatric and parents’ attitudes towards COVID-19 vaccines and intention to vaccinate for children. *Journal of Korean Medical Science*.

[42] Hetherington E., Edwards S. A., MacDonald S. E. (2021). SARS-CoV-2 vaccination intentions among mothers of children aged 9 to 12 years: a survey of the All Our Families cohort. *CMAJ Open*.

[43] Ruggiero K. M., Wong J., Sweeney C. F. (2021). Parents’ intentions to vaccinate their children against COVID-19. *Journal of Pediatric Health Care*.

[44] Teasdale C. A., Borrell L. N., Shen Y. (2021). Parental plans to vaccinate children for COVID-19 in New York city. *Vaccine*.

[45] Valladares-Garrido M. J., Picón-Reátegui C. K., Zila-Velasque J. P., Grados-Espinoza P. (2022). Prevalence and factors associated with insomnia in military personnel: a retrospective study during the second COVID-19 epidemic wave in Peru. *Healthcare*.

[46] Díaz-Vélez C., Failoc-Rojas V. E., Valladares-Garrido M. J. (2021). SARS-CoV-2 seroprevalence study in Lambayeque, Peru. June–July 2020. *PeerJ*.

[47] Campbell-Sills L., Stein M. B. (2007). Psychometric analysis and refinement of the connor–davidson resilience scale (CD-RISC): validation of a 10-item measure of resilience. *Journal of Traumatic Stress*.

[48] Dolores Serrano-Parra M., Garrido-Abejar M., Notario-Pacheco B., Bartolomé-Gutiérrez R., Solera-Martínez M., Martínez-Vizcaíno V. (2013). Validity of the Connor-Davidson resilience scale (10 items) in a population of elderly. *Enfermería Clínica*.

[49] Blanco V., Guisande M. A., Sánchez M. T., Otero P., Vázquez F. L. (2019). Spanish validation of the 10-item connor–davidson resilience scale (CD-RISC 10) with non-professional caregivers. *Aging and Mental Health*.

[50] Notario-Pacheco B., Solera-Martínez M., Serrano-Parra M. D., Bartolomé-Gutiérrez R., García-Campayo J., Martínez-Vizcaíno V. (2011). Reliability and validity of the Spanish version of the 10-item Connor-Davidson Resilience Scale (10-item CD-RISC) in young adults. *Health and Quality of Life Outcomes*.

[51] Notario-Pacheco B., Martínez-Vizcaíno V., Trillo-Calvo E., Pérez-Yus M. C., Serrano-Parra D., García-Campayo J. (2014). Validity and reliability of the Spanish version of the 10-item CD-RISC in patients with fibromyalgia. *Health and Quality of Life Outcomes*.

[52] Spitzer R. L., Kroenke K., Williams J. B. W., Löwe B. (2006). A brief measure for assessing generalized anxiety disorder: the GAD-7. *Archives of Internal Medicine*.

[53] Mills S. D., Fox R. S., Malcarne V. L., Roesch S. C., Champagne B. R., Sadler G. R. (2014). The psychometric properties of the generalized anxiety disorder-7 scale in Hispanic Americans with English or Spanish language preference. *Cultural Diversity and Ethnic Minority Psychology*.

[54] Mossman S. A., Luft M. J., Schroeder H. K. (2017). The Generalized Anxiety Disorder 7-item scale in adolescents with generalized anxiety disorder: signal detection and validation. *Annals of Clinical Psychiatry*.

[55] Spitzer R. L., Kroenke K., Williams J. B. W. (1999). Validation and utility of a self-report version of PRIME-MDThe PHQ primary care study. *JAMA*.

[56] Kroenke K., Spitzer R. L., Williams J. B. W. (2001). The PHQ-9. *Journal of General Internal Medicine*.

[57] Villarreal-Zegarra D., Copez-Lonzoy A., Bernabé-Ortiz A., Melendez-Torres G. J., Bazo-Alvarez J. C. (2019). Valid group comparisons can be made with the Patient Health Questionnaire (PHQ-9): a measurement invariance study across groups by demographic characteristics. *PLoS One*.

[58] Olivares-Faúndez V. e., Mena-Miranda L., Macía-Sepúlveda F., Jélvez-Wilke C. (2014). Validez factorial del Maslach Burnout Inventory Human Services (MBI-HSS) en profesionales chilenos. *Universitas Psychologica*.

[59] Maticorena-Quevedo J., Beas R., Anduaga-Beramendi A., Mayta-Tristán P. (2016). Prevalencia del síndrome de burnout en médicos y enfermeras del Perú, ENSUSALUD 2014. *Revista Peruana de Medicina Experimental y Salud Pública*.

[60] Miles J. N. V., Marshall G. N., Schell T. L. (2008). Spanish and English versions of the PTSD Checklist–Civilian version (PCL-C): testing for differential item functioning. *Journal of Traumatic Stress*.

[61] Vera-Villarroel P., Celis-Atenas K., Córdova-Rubio N., Zych I., Buela-Casal G. (2011). Chilean validation of the posttraumatic stress disorder checklist–civilian version (pcl–C) after the earthquake on february 27, 2010. *Psychological Reports*.

[62] Flores E. C., Carnero A. M., Bayer A. M. (2014). Social capital and chronic post-traumatic stress disorder among survivors of the 2007 earthquake in Pisco, Peru. *Social Science and Medicine*.

[63] Rubio G., Montero J., Jáuregui J. (1998). Validacion de la escale de riesgo suicida de Plutchik en poblacion espanola. *Archivos de Neurobiologia*.

[64] Santana-Campas M. A., Santoyo Telles F. (2018). Propiedades psicométricas de la escala riesgo suicida de Plutchik en una muestra de jóvenes mexicanos privados de la libertad. *Avances En Psicología*.

[65] Yuly S. C., Palacio-Sañudo J., Caballero-Domínguez C. C., Pineda-Roa C. A. (2019). Adaptación, validez de constructo y confiabilidad de la escala de riesgo suicida Plutchik en adolescentes colombianos. *Revista Latinoamericana de Psicología*.

[66] Zhang H., Zheng P., Zhang J. (2021). Vaccine hesitancy among parents and its influencing factors: a cross-sectional study in Guangzhou, China. *Human Vaccines and Immunotherapeutics*.

[67] Bagateli L. E., Saeki E. Y., Fadda M., Agostoni C., Marchisio P., Milani G. P. (2021). COVID-19 vaccine hesitancy among parents of children and adolescents living in Brazil. *Vaccines*.

[68] Horiuchi S., Sakamoto H., Abe S. K. (2021). Factors of parental COVID-19 vaccine hesitancy: a cross sectional study in Japan. *PLoS One*.

[69] Alfieri N. L., Kusma J. D., Heard-Garris N. (2021). Parental COVID-19 vaccine hesitancy for children: vulnerability in an urban hotspot. *BMC Public Health*.

[70] Seiler M., Goldman R. D., Staubli G., Hoeffe J., Gualco G., Manzano S. (2022). Parents’ intent to vaccinate against influenza during the COVID-19 pandemic in two regions in Switzerland. https://smw.ch/article/doi/smw.2021.20508.

[71] Skjefte M., Ngirbabul M., Akeju O. (2021). COVID-19 vaccine acceptance among pregnant women and mothers of young children: results of a survey in 16 countries. *European Journal of Epidemiology*.

[72] Rajmil L., Hjern A., Boran P., Gunnlaugsson G., Kraus de Camargo O., Raman S. (2021). Impact of lockdown and school closure on children’s health and well-being during the first wave of COVID-19: a narrative review. *BMJ Paediatrics Open*.

[73] Viner R. M., Russell S. J., Croker H. (2020). School closure and management practices during coronavirus outbreaks including COVID-19: a rapid systematic review. *The Lancet Child and Adolescent Health*.

[74] Yigit M., Ozkaya-Parlakay A., Senel E. (2021). Evaluation of COVID-19 vaccine refusal in parents. *The Pediatric Infectious Disease Journal*.

[75] Murphy J., Vallières F., Bentall R. P. (2021). Psychological characteristics associated with COVID-19 vaccine hesitancy and resistance in Ireland and the United Kingdom. *Nature Communications*.

[76] Fond G., Nemani K., Etchecopar-Etchart D. (2021). Association between mental health disorders and mortality among patients with COVID-19 in 7 countries: a systematic review and meta-analysis. *JAMA Psychiatry*.

[77] Nguyen K. H., Chen S., Morris K., Chui K., Allen J. D. (2022). Mental health symptoms and association with COVID-19 vaccination receipt and intention to vaccinate among adults, United States. *Preventive Medicine*.

[78] Abdel Wahed W. Y., Hefzy E. M., Ahmed M. I., Hamed N. S. (2020). Assessment of knowledge, attitudes, and perception of health care workers regarding COVID-19, A cross-sectional study from Egypt. *Journal of Community Health*.

[79] Temsah M.-H., Alhuzaimi A. N., Aljamaan F. (2021). Parental attitudes and hesitancy about COVID-19 vs. Routine childhood vaccinations: a national survey. *Frontiers in Public Health*.

[80] Milan S., Dáu A. L. B. T. (2021). The role of trauma in mothers’ COVID-19 vaccine beliefs and intentions. *Journal of Pediatric Psychology*.

[81] Corcoran K. E., Scheitle C. P., DiGregorio B. D. (2021). Christian nationalism and COVID-19 vaccine hesitancy and uptake. *Vaccine*.

[82] Lacsa J. E. M. (2021). COVID-19 vaccination program: the Catholic Church’s all-time support to the government when it is for the common good. *Journal of Public Health*.

[83] Williams J. T. B., Rice J. D., O’Leary S. T. (2021). Associations between religion, religiosity, and parental vaccine hesitancy. *Vaccine X*.

[84] Whitehead A. L., Perry S. L. (2020). How culture wars delay herd immunity: christian nationalism and anti-vaccine attitudes. *Socius*.

[85] Yao H., Chen J.-H., Xu Y.-F. (2020). Patients with mental health disorders in the COVID-19 epidemic. *The Lancet Psychiatry*.

[86] Oppenauer C., Burghardt J., Kaiser E., Riffer F., Sprung M. (2021). Psychological distress during the COVID-19 pandemic in patients with mental or physical diseases. *Frontiers in Psychology*.

